# Can heightened aesthetic sensitivity in music promote empathy?-A pre- and post-test multilevel linear model of an 8-week music aesthetic education course intervention

**DOI:** 10.3389/fpsyg.2026.1744584

**Published:** 2026-04-08

**Authors:** Mingyang Xia, Jiali Li, Xin Li, Ziyan Zhang

**Affiliations:** 1 Department of Art and Music Didactics, Universitat de València, Valencia, Spain; 2 Institute of International Cooperation and Local Development, Universitat de València, Valencia, Spain; 3 School of Mathematics and Statistics, Chongqing Technology and Business University, Chongqing, China; 4 School of Humanities, Guangxi Arts University, Nanning, China

**Keywords:** aesthetic sensitivity, empathy, mediating effect, multilevel linear model, music aesthetic education, quasi-experiment

## Abstract

**Introduction:**

Aesthetic education is expected to support non-cognitive development in higher education, but whether music aesthetic education can improve empathy and whether aesthetic sensitivity (AS) mediates this effect remain unclear.

**Methods:**

We conducted an 8-week whole-class quasi-experiment with 208 undergraduates from two intact classes at two Chinese universities. AS and empathy were measured before (T0) and after (T1) the intervention. Given class nesting, primary analyses used multilevel linear modeling, with ANCOVA and inverse probability weighting as robustness checks. Within the intervention class, we also tested whether changes in AS mediated empathy gains.

**Results:**

Within the experimental class, pre-post empathy gains were associated with gains in AS, in a pattern consistent with an indirect pathway through AS. Empathy also increased significantly within the intervention class, while between-class post-test differences were positive but not uniformly significant. Multilevel models showed that AS was positively associated with empathy. Mediation analyses indicated that increases in AS statistically accounted for part of the observed empathy gains.

**Discussion:**

These findings suggest that curriculum-based music aesthetic education may support empathy development in higher education and that heightened AS may be one mechanism linking the intervention to empathy outcomes. Given the quasi-experimental design, the small number of clusters, and the self-report measures, stronger designs and larger samples are needed to confirm causal effects.

## 1 Introduction

This study focuses on a practical and testable question in higher education: Can a curriculum-based music aesthetic education course improve college students' empathy, and does heightened aesthetic sensitivity (AS) help explain this change? In this paper, music aesthetic education refers to structured teaching that trains students to perceive, interpret, and evaluate musical form and expressive meaning through guided listening, analysis, discussion, and reflective tasks. This is different from (a) passive music exposure (simply listening without guided work) and (b) skill-based performance training that primarily targets technique. Prior work suggests that music experience may relate to empathy, but much of the evidence is still correlational or observational, definitions and operationalizations vary across studies, and mechanistic tests remain comparatively scarce (Davila-Barrio et al., 2023; Morand-Grondin et al., 2025; Cooper, 2026). To address this gap, we examine AS as a theoretically grounded mediating mechanism, while acknowledging that experimental evidence on music-induced empathy is emerging (e.g., McDonald et al., 2022).

This definition is consistent with Reimer's aesthetic view that music education should make the distinctive meanings and values of musical sounds widely and deeply available through direct, guided experience that is deepened by skills, knowledge, attitudes, and cultivated sensitivities (Reimer, 2003; Lines, 2022; Zhao et al., 2025). In Reimer's account, aesthetic education in music aims to enhance learning that (a) treats musical sounds as sources of shareable meanings, (b) involves an integration of mind, body, and feeling, and (c) requires direct experience with musical sounds supported by education-cultivated skills and sensitivities (Reimer, 2003; Lines, 2022; Zhao et al., 2025). AS is a multidimensional construct involving perception, cognition, and emotion. It refers to an individual's ability to perceive, interpret, and evaluate aesthetic qualities in artworks or other stimuli (Corradi et al., 2020; Clemente et al., 2022; Friedman et al., 2024). In music education, AS captures sensitivity to musical structure and expressive cues that can be trained through guided listening and reflection (Barrett, 2002; Lines, 2022; Zhao et al., 2025).

Among the various modalities of aesthetic education, music education has been proposed as a pathway for empathy development because it can communicate and evoke emotion across linguistic and cultural boundaries (Fritz et al., 2009; Sievers et al., 2013; Davila-Barrio et al., 2023; Cooper, 2026). Empathy comprises cognitive (e.g., perspective taking) and affective (e.g., emotional sharing) dimensions, and music may engage both through emotional resonance, narrative imagery, and shared attention. Recent reviews and syntheses highlight that the evidence base is heterogeneous: definitions and measures vary, many studies are correlational, and findings are sometimes mixed (Davila-Barrio et al., 2023; Morand-Grondin et al., 2025; Cooper, 2026). Experimental work indicates that emotionally evocative music can acutely enhance empathic responding and prosocial decisions (McDonald et al., 2022), yet curriculum-based interventions in higher education that test both outcomes and mechanisms remain comparatively rare (Morand-Grondin et al., 2025).

The present study proposes the core construct of aesthetic sensitivity (AS) as a mediator between music aesthetic education and empathy. In line with calls for authentic, curriculum-based pedagogy, we implemented an 8-week quasi-experimental intervention in natural classroom settings. Using a pre-post design and multilevel modeling (HLM), we investigated: (1) if the intervention increased college students' AS; (2) if it increased cognitive/emotional empathy; and (3) if AS mediated the relationship between music education and empathy.

Building on this literature, we focus on two under-addressed issues in higher education: first, whether a curriculum-embedded, classroom-based music aesthetic education course can produce measurable gains in empathy beyond correlational associations; and second, whether aesthetic sensitivity (AS) functions as a theoretically proximal mechanism linking aesthetic learning to empathy outcomes. Addressing these issues helps clarify where, when, and how aesthetic-oriented music pedagogy may contribute to students' social–emotional development.

Accordingly, we test three hypotheses. H1: after controlling for baseline levels, students in the intervention class will show higher post-test empathy than students in the control class. H2: the intervention will increase students' AS. H3: changes in AS will statistically mediate the association between the intervention and empathy outcomes.

## 2 Methods

This section describes the study design, participants and procedure, measures, and analytic strategy used to examine whether an 8-week music aesthetic education course intervention improves aesthetic sensitivity and empathy, and whether changes in AS statistically mediate empathy gains.

### 2.1 Research design

This study used a whole-class quasi-experimental design, a common approach for evaluating curriculum-based interventions in authentic educational settings (Gopalan et al., 2020; Völlinger et al., 2023). Specifically, we recruited 216 undergraduate students from two intact general-education classes in two institutions. The two classes served as an intervention class and a control class. Based on student availability and questionnaire completeness, 208 paired pre- and post-test questionnaires were retained (response rate = 96.3%).

Setting and ethics. The intervention and data collection were conducted in Nanning (Guangxi) and Chongqing, China in regular classroom settings. This study involved human participants in a classroom-based educational intervention and anonymous questionnaire survey. Before the pre-test, students received an information sheet explaining the study purpose, procedures, anonymity, and the voluntary nature of participation. Written informed consent was obtained from all participants, and no identifying information was collected. Students could decline or withdraw at any time without penalty.

We collected outcomes at two time points: T0 (pre-test) and T1 (post-test, immediately after the 8-week course). Before the intervention, all participants completed baseline measures of AS and empathy. The same measures were administered at T1 to capture change. Because students were nested within intact classes, the primary analysis used multilevel modeling (hierarchical linear modeling) to account for clustering and to estimate the intervention effect (Khine, 2022). As robustness checks, we also estimated covariate-adjusted ANCOVA models and applied inverse probability weighting to address observed confounding and potential imbalance (Bailey et al., 2023; Hu et al., 2025).

#### 2.1.1 Intervention and instructional consistency

The intervention was implemented as an 8-week music aesthetic education course embedded in regular classroom teaching. Each week combined guided listening to selected works, teacher-led analysis of musical elements and expressive cues, short group discussion, and brief written reflection. In-class tasks asked students to attend to and evaluate formal beauty, emotional expression, technical ability, and artistic value—classroom dimensions relevant to the aesthetic sensitivity construct assessed in this study. The weekly sequence progressed from basic musical elements (Week 1) and emotional cues (Week 2), to musical styles and cultural context (Week 3), aesthetic experience and emotion regulation (Week 4), collaborative musical practice (Week 5), musical narrative and meaning interpretation (Week 6), comprehensive aesthetic reflection and migration (Week 7), and a performance/summary activity (Week 8). The detailed weekly schedule and examples are provided in the Supplementary Appendix 1. To support comparability across classes, the intervention followed the same week-by-week plan and assessment schedule (T0/T1) with identical questionnaire instructions.

Table 1 summarizes participants' demographic and music-exposure characteristics. Briefly, the two intact classes contributed similar numbers of students, the gender distribution was approximately balanced, and most participants were 20–21 years old. Majors were diverse across liberal arts, science, engineering, and arts-related fields. Prior music-learning experience and everyday music exposure were also heterogeneous, which supports the use of covariates in subsequent models rather than item-by-item narrative repetition of Table 1.

**Table 1:** Frequency statistics of basic population information.

Basic information		Frequency	Percentage
School	A	100	48.08%
	B	108	51.92%
Class	1	100	48.08%
	2	108	51.92%
Gender	Male	103	49.52%
	Female	105	50.48%
Age	20 years	109	52.40%
	21 years	99	47.60%
Field of Study	Humanities	73	35.10%
	Sciences	53	25.48%
	Engineering	49	23.56%
	Arts	25	12.02%
	Other	8	3.85%
Music Learning Experience	None	45	21.63%
	Less than 1 year	99	47.60%
	1-3 years	39	18.75%
	More than 3 years	25	12.02%
Usual Frequency of Engaging with Music	Nearly every day	21	10.10%
	Several times per week	40	19.23%
	Several times per month	112	53.85%
	Rarely or never	35	16.83%
Total		208	100%

### 2.2 Variable description

Aesthetic sensitivity (AS) was measured using the Aesthetic Sensitivity Scale (ASS; Abdolmaleki, 2013), a 34-item self-report instrument with three subscales: Sensitivity to Natural Beauty (NBS), Sensitivity to Artistic Beauty (ABS), and Sensitivity to Emotion and Resonance (ES). Items were rated on a 7-point Likert scale (1 = completely disagree; 7 = completely agree), with higher scores indicating higher AS. The ASS continues to be used in recent psychometric and applied research (e.g., Bayat et al., 2022). Empathy was measured using the Chinese version of the Interpersonal Reactivity Index (C-IRI; Davis, 1980, 1983; Huang et al., 2012), which includes four subscales: Perspective Taking (PT) and Fantasy/Imagination (both reflecting cognitive empathy), and Empathic Concern (EC) and Personal Distress (PD) (reflecting affective empathy). Responses were recorded on a 5-point Likert scale (1 = completely disagree; 5 = completely agree), with higher scores indicating higher empathy. The C-IRI remains widely used in contemporary Chinese samples (e.g., Li et al., 2024; Song et al., 2025) and is also commonly used as a convergent measure in recent psychometric validation work (e.g., Ge et al., 2023). Control variables included gender, age, major category, prior music learning experience, and usual frequency of engaging with music. All questionnaires were administered at T0 and T1 after participants listened to selected music excerpts in a quiet classroom, and attention-check items were used to improve data quality.

#### 2.2.1 Reliability and validity of measures

As reported in Table 2, internal-consistency indices for the ASS subscales and the total empathy scales were acceptable, supporting their use for the subsequent pre–post comparisons and multilevel analyses.

**Table 2:** Reliability test.

Dimension	Items	Cronbach's α
Sensitivity to Natural Beauty	12	0.882
Sensitivity to Artistic Beauty	12	0.87
Sensitivity to Emotion & Resonance	10	0.842
Aesthetic Sensitivity Scale (total)	34	0.951
Empathy Scale (total)	22	0.932

Construct validity was examined using standard factorability diagnostics: the Kaiser–Meyer–Olkin (KMO) measure of sampling adequacy and Bartlett's test of sphericity (Kaiser, 1974; Bartlett, 1950). Higher KMO values and a significant Bartlett test indicate that the correlation matrix is suitable for factor analysis and that meaningful latent structure can be extracted.

In this study, the KMO value was 0.957 and Bartlett's test of sphericity was significant (*p* < 0.001) (Table 3), indicating that the data were appropriate for factor analysis and that the scale demonstrated good construct validity.

**Table 3:** Results of KMO and Bartlett's Test of Sphericity.

KMO measure of sampling adequacy	0.957	
Bartlett's Test of Sphericity, Approx.	*χ^2^*	5739.918
	*df*	1,540
	*p*	<0.001

## 3 Results

Analyses were conducted in IBM SPSS Statistics 26.0. Results are reported in three steps aligned with the hypotheses: preliminary checks (baseline equivalence and common-method diagnostics), intervention effects on AS and empathy (between-class post-test comparisons and within-class pre–post changes), and multilevel and mediation models testing AS as a mechanism.

### 3.1 Preliminary analyses and baseline equivalence

At T0, the intervention and control classes did not differ significantly on AS or empathy indicators (Table 4; all *p* > 0.05). For empathy, independent-samples tests showed no baseline differences for cognitive empathy (*t* = 0.501, *p* = 0.617), emotional empathy (*t* = 0.678, *p* = 0.498), or total empathy (*t* = 0.612, *p* = 0.541). These results support baseline comparability and motivate the subsequent analyses of post-test differences and pre–post change.

**Table 4:** Group comparisons between the experimental class and the control class in the pre-test.

Variable	Group	Mean	Standard deviation	*t*	*p*	Cohen's *d*
Sensitivity to Natural Beauty (NBS)	Experimental Group	3.03	0.94	−0.204	0.838	0.028
	Control Group	3.06	0.91			
Sensitivity to Artistic Beauty (ABS)	Experimental Group	3.19	0.97	0.254	0.8	0.035
	Control Group	3.15	0.86			
Sensitivity to Emotion and Resonance (ES)	Experimental Group	3.21	0.9	0.53	0.597	0.074
	Control Group	3.14	0.93			
Aesthetic Sensitivity	Experimental Group	3.14	0.9	0.2	0.841	0.028
	Control Group	3.12	0.86			
Cognitive Empathy	Experimental Group	2.39	0.79	0.501	0.617	0.07
	Control Group	2.34	0.74			
Emotional Empathy	Experimental Group	2.38	0.78	0.678	0.498	0.094
	Control Group	2.31	0.75			
Empathy as a whole	Experimental Group	2.39	0.76	0.612	0.541	0.085
	Control Group	2.32	0.72			

As an additional diagnostic, Harman's single-factor test suggested that common method variance was unlikely to be severe: the first unrotated factor explained 38.491% of the total variance, below the conventional 40% threshold (Podsakoff et al., 2024). This check is descriptive rather than definitive and is interpreted cautiously.

### 3.2 Intervention effects on AS and empathy

Between-class post-test comparisons (Table 5) indicated higher mean AS scores (NBS, ABS, and overall AS) in the intervention class than in the control class, with effects in the expected direction but not consistently reaching conventional statistical significance (*p* > 0.05 for some contrasts). Empathy post-test means were also slightly higher in the intervention class, again with between-class differences that were positive but not uniformly statistically significant.

**Table 5:** Group comparisons between the experimental class and the control class in the post-test.

Variable	Group	Mean	Standard deviation	*t*	*p*	Cohen's *d*
Sensitivity to Natural Beauty (NBS)	Experimental Group	5.35	1.19	−1.417	0.158	0.199
	Control Group	5.14	0.88			
Sensitivity to Artistic Beauty (ABS)	Experimental Group	5.48	1.13	−1.203	0.23	0.168
	Control Group	5.3	0.93			
Sensitivity to Emotion and Resonance (ES)	Experimental Group	5.48	1.2	−1.536	0.126	0.315
	Control Group	5.26	0.9			
Aesthetic Sensitivity	Experimental Group	5.44	1.14	−1.439	0.152	0.202
	Control Group	5.23	0.86			
Cognitive Empathy	Experimental Group	3.91	0.85	−1.66	0.099	0.233
	Control Group	3.77	0.64			
Emotional Empathy	Experimental Group	3.87	0.78	−1.789	0.075	0.25
	Control Group	3.69	0.66			
Empathy as a whole	Experimental Group	3.89	0.79	−1.792	0.075	0.251
	Control Group	3.72	0.62			

Within the intervention class, paired comparisons showed clear pre–post gains (Table 6). AS increased from T0 to T1 across NBS, ABS, and ES, with statistically significant paired tests (*p* < 0.001). Empathy also increased from T0 to T1 for cognitive empathy, emotional empathy, and total empathy, indicating that changes were most evident within the treated classroom context.

**Table 6:** Pre-post changes in the experimental class.

Variable	Time point	Mean	Standard deviation	*t*	*p*	Cohen's *d*
Sensitivity to Natural Beauty (NBS)	Pre-test	3.03	0.94	−13.907	< 0.001	1.967
	Post-test	5.35	1.19			
Sensitivity to Artistic Beauty (ABS)	Pre-test	3.19	0.97	−14.212	< 0.001	2.01
	Post-test	5.48	1.13			
Sensitivity to Emotion and Resonance (ES)	Pre-test	3.21	0.9	−13.712	< 0.001	1.939
	Post-test	5.48	1.2			
Aesthetic Sensitivity	Pre-test	3.14	0.9	−14.476	< 0.001	2.047
	Post-test	5.44	1.14			
Cognitive Empathy	Pre-test	2.39	0.79	−11.61	< 0.001	1.642
	Post-test	3.91	0.85			
Emotional Empathy	Pre-test	2.38	0.78	−11.852	< 0.001	1.676
	Post-test	3.87	0.78			
Empathy as a whole	Pre-test	2.39	0.76	−12.127	< 0.001	1.715
	Post-test	3.89	0.79			

Pearson correlations (Table 7) indicated positive associations among the AS dimensions and empathy indicators; most correlations were statistically significant (*p* < 0.01). These bivariate relationships provide descriptive support for the multilevel and mediation models reported below, which account for class nesting and covariates.

**Table 7:** Correlation analysis.

Variable	Sensitivity to Natural Beauty (NBS)	Sensitivity to Artistic Beauty (ABS)	Sensitivity to Emotion and Resonance (ES)	Cognitive empathy	Emotional empathy
Sensitivity to Natural Beauty (NBS)	1				
Sensitivity to Artistic Beauty (ABS)	0.950^**^	1			
Sensitivity to Emotion and Resonance (ES)	0.952^**^	0.934^**^	1		
Cognitive Empathy	0.936^**^	0.921^**^	0.921^**^	1	
Emotional Empathy	0.942^**^	0.941^**^	0.928^**^	0.924^**^	1

^*^p < 0.05;

^**^p < 0.01.

### 3.3 Multilevel model estimates

Multilevel models were estimated to account for the nesting of students within intact classes and to test whether AS predicts empathy after accounting for clustering and student covariates (Table 8).

**Table 8:** Fixed Effects and Random Effects Table.

Variable		Fixed effects		Variance explained
		Coefficient	Standard error	
Model 1	Intercept	0.035	0.037	12.13%
	Gender	0.044	0.037	
	Age	−0.067	0.037	
	Field of Study	−0.014	0.015	
	Music-Learning Experience (Yes/No)	0.019	0.02	
	Frequency of Music Exposure	0.016	0.022	
Model 2	Aesthetic Sensitivity	0.676^**^	0.012	50.10%

^*^p < 0.05;

^**^p < 0.01.

The unconditional (null) model yielded an intraclass correlation coefficient, ICC(1) = 0.104, indicating that approximately 10% of the variance in empathy was attributable to between-class differences. This level of clustering supports multilevel modeling rather than single-level regression.

The Level-1 (student-level) model is:


$$\begin{array}{l} Empathy_{ij}=\beta_{0j}+\beta 1\left(Gender_{ij}\right)+\beta_{2}\left(Age_{ij}\right)+\beta_{3}\left(Major_{ij}\right)\\ \;+\beta_{4}\left(MusicExp_{ij}\right)+\beta 5\left(MusicFreq_{ij}\right)+r_{ij} \end{array}$$

The full model parameters are as follows:


$$\begin{array}{l} Empathy_{ij}=\beta_{0j}+\beta_{1}\left(Gender_{ij}\right)+\beta_{2}\left(Age_{ij}\right)+\beta_{3}\left(Major_{ij}\right)\\ \;+\beta_{4}\left(MusicExp_{ij}\right)+\beta_{5}\left(MusicFreq_{ij}\right)\\ \;+\beta_{6}\left(AestheticSensitivity_{ij}\right)+r_{ij} \end{array}$$

The Level-2 (class-level) model (random intercept; slopes fixed) is:


$$\begin{array}{l} \beta_{0j}=\gamma_{00}+\;\mu_{0j}\\ \beta_{1j}=\;\gamma_{10}\\ \beta_{2j}=\;\gamma_{20}\\ \beta_{3j}=\;\gamma_{30}\\ \beta_{4j}=\;\gamma_{40}\\ \beta_{5j}=\;\gamma_{50}\\ \beta_{6j}=\;\gamma_{60} \end{array}$$

Among them, Empathy_*ij*_ is the level of empathy of the i-th student in the j-th class; β_0*j*_ is the class-level intercept term; r_*ij*_ is the student-level random error term; γ00 is the overall mean empathy score; μ_0*j*_ is the class-level random effect.

Model 1 added student background covariates (gender, age, major category, prior music-learning experience, and frequency of daily music exposure). None of these covariates showed a statistically significant association with empathy in this sample (all *p* > 0.05), suggesting limited explanatory power of baseline background variables for empathy outcomes (Table 8).

Model 2 then included AS as the focal predictor. AS showed a significant positive association with empathy (*B* = 0.676, *SE* = 0.012, *p* < 0.001). Substantively, a one-unit increase in AS corresponded to an estimated 0.676-unit increase in empathy. The model accounted for a substantial proportion of variance (reported explanatory rate = 50.10%; Table 8).

Overall, the multilevel results converge with the bivariate correlations and support AS as a meaningful predictor of empathy while appropriately modeling class-level clustering.

### 3.4 Mediating effect analysis results

Taken together, these results are consistent with an indirect pathway through AS underlying the observed pre-post increase in empathy within the experimental class, coding measurement occasion as Time (T0 = 0, T1 = 1) and estimating the total effect (Time → Empathy), the a-path (Time → AS), and the joint model including both predictors (Table 9).

**Table 9:** Mediation analysis of pre–post change within the experimental class.

Predictor		Empathy			Aesthetic sensitivity			Empathy		
		*B*	*t*	*p*	*B*	*t*	*p*	*B*	*t*	*p*
Constant		0.898	7.994	< 0.001	0.917	6.272	< 0.001	0.239	5.727	< 0.001
Independent variable	Time (T1 vs T0)	1.455	20.484	< 0.001	2.211	23.923	< 0.001	−0.133	−3.423	0.001
Mediating variable	Aesthetic Sensitivity							0.718	53.478	< 0.001
Model fit	*R^2^*	0.503			0.58			0.937		
	Adjusted *R^2^*	0.502			0.579			0.937		
	*F* Value	419.581			572.316			3088.508		

First, Time significantly predicted empathy (*B* = 1.455, *t* = 20.484, *p* < 0.001), indicating a clear pre–post increase in empathy within the intervention class. Second, Time significantly predicted AS (*B* = 2.211, *t* = 23.923, *p* < 0.001), consistent with the intended effect of the aesthetic-education curriculum on students' sensitivity to musical form and expression.

Third, when both Time and AS were entered simultaneously, AS significantly predicted empathy (*B* = 0.718, *t* = 53.478, *p* < 0.001), and overall model fit was strong (*R*^2^ = 0.937; *F* = 3088.508, *p* < 0.001). Notably, because AS and empathy are highly correlated, the remaining direct effect of Time is small and should be interpreted cautiously. Taken together, these results provide statistical support for AS as a mediating mechanism underlying the observed empathy increase, consistent with H3.

## 4 Discussion

This discussion synthesizes the main findings from the 8-week whole-class quasi-experiment and situates them within recent literature on music, aesthetics, and empathy. Because intact classes were assigned to conditions, we interpret effects cautiously: baseline equivalence checks and covariate adjustment reduce (but cannot fully eliminate) class-level confounding. We therefore emphasize patterns that are consistent across analytic approaches, highlight plausible alternative explanations, and specify concrete design features and measurement strategies for future studies.

### 4.1 Interpretation and alternative explanations

Because intact classes were assigned to intervention vs. control conditions, the design cannot fully eliminate class-level confounding (e.g., class climate, instructor style, or student composition). Baseline equivalence checks and statistical controls reduce but do not remove this concern. In addition, empathy gains could reflect broader factors such as general classroom engagement, instructor enthusiasm, or increased peer interaction during collaborative activities, rather than aesthetic sensitivity alone. We therefore adopt cautious causal language and present AS as a plausible mechanism that warrants further testing in designs with stronger causal leverage.

An additional consideration is measurement reactivity: repeated exposure to the same self-report scales may increase respondents' awareness of the constructs and contribute to modest score inflation. However, baseline equivalence, the inclusion of covariates, and the convergence of within-class change with the multilevel and mediation results jointly reduce (but do not eliminate) this concern. Future studies could strengthen causal attribution by randomizing more classes, adding an active control condition, and including behavioral empathy indicators alongside self-report measures.

### 4.2 Effects of music aesthetic education on AS and empathy

After 8 weeks, the experimental group scored higher than controls on the ASS, indicating that systematic guided listening and reflection can strengthen aesthetic sensitivity. This is consistent with recent empirical work conceptualizing music aesthetic literacy as a learnable, multidimensional outcome in non-music majors (Zhao et al., 2025) and with broader discussions of aesthetics as a core dimension of music education (Lines, 2022). Empathy also improved in the intervention class, particularly in affective empathy.

Notably, the pattern of statistical significance differed by analytic lens: within-class pre–post gains in the intervention class were consistently significant, whereas between-class post-test contrasts were positive but sometimes non-significant. This pattern is common in classroom-based quasi-experiments with a small number of clusters and suggests that the intervention effect is most clearly observed as change over time within the treated instructional context. More broadly, evidence syntheses indicate that the social–emotional benefits of music participation are plausible but uneven, with substantial heterogeneity in intervention types, definitions, and outcome measures (Davila-Barrio et al., 2023; Cooper, 2026).

### 4.3 Psychological mechanisms linking AS and empathy

The mediation analyses suggest that increases in AS help explain empathy gains through an indirect pathway. This pattern is consistent with recent work arguing that evidence for a simple “music training → empathy” pathway is limited and that more mechanistic testing is needed to specify which components of empathy change and under what conditions (Morand-Grondin et al., 2025). Conceptually, engaging with musical expressive cues provides repeated practice in affective attunement and perspective taking, which may generalize to interpersonal contexts (Davila-Barrio et al., 2023). Relatedly, emotionally evocative music can produce short-term increases in empathic responding and prosocial decisions, but such effects do not automatically generalize to all domains of social cognition (McDonald et al., 2022).

Mechanistically, the course activities (guided listening, meaning interpretation, and reflective discussion) may cultivate attentional sensitivity to expressive cues and promote emotional resonance, which are proximal skills for empathic engagement. This interpretation aligns with reviews emphasizing the role of interactional and ensemble contexts in empathy development (Davila-Barrio et al., 2023).

### 4.4 Theoretical contributions and practical implications

Methodologically, the study extends intervention evidence in higher education by using multilevel models to account for class nesting (Gopalan et al., 2020; Khine, 2022). Substantively, the findings add to emerging work on aesthetic education outcomes in university students (Ye et al., 2025) and complement empathy-focused social and emotional learning (SEL) evaluations demonstrating that empathy can be cultivated through structured curricula (Silke et al., 2024). Within higher education, such non-cognitive capacities are increasingly framed as central to holistic student development (Shek et al., 2023). Conceptually, by focusing on AS as an intermediate outcome, the study helps specify which aspect of musical learning may plausibly connect to empathy, addressing calls for more mechanistic accounts in this domain. This framing also resonates with contemporary discussions of aesthetics as a core educational aim that can be taught and assessed rather than treated as a purely subjective byproduct (Lines, 2022).

In practice, the findings suggest that empathy-oriented music aesthetic education may benefit from three design features: (1) structured guided listening that explicitly trains attention to musical form and expressive cues; (2) regular reflection prompts that ask students to connect musical meaning to emotions and social situations; and (3) collaborative activities (e.g., chorus/ensemble tasks) that require listening-and-responding to peers. Assessment can be aligned with the three ASS dimensions used here by using simple rubrics for formal beauty, emotional expression, technical ability, and artistic value, combined with brief reflective writing.

### 4.5 Limitations

Several limitations should be noted. First, the quasi-experimental assignment of intact classes limits causal inference relative to randomized trials and may leave residual confounding (Cook and Campbell, 1979; Shadish et al., 2002). Second, the sample was limited to Chinese non-music majors in two institutions, which constrains external validity and generalizability to other populations and contexts (Henrich et al., 2010). Third, the 8-week window precludes conclusions about the durability of changes; longer-term follow-up is needed to evaluate sustainability and potential attenuation of effects (Cook and Campbell, 1979). Finally, AS and empathy were measured via self-report, which can be influenced by social desirability and common method bias despite procedural remedies (Crowne and Marlowe, 1960; Podsakoff et al., 2024). Future studies should consider behavioral or performance-based empathy measures and randomized or matched cluster designs with more classes.

In addition, the study relied on only two classes, which constrains the stability of multilevel variance estimates (including ICC) and limits the ability to test class-level moderators. Replication with more clusters is needed to confirm the magnitude of between-class variation and to evaluate whether instructional features (e.g., teacher behaviors or classroom climate) condition the intervention–outcome relationship.

Third, the correlations between AS and empathy dimensions were very high (Table 7), and the mediation model explained a large proportion of variance. Because both constructs were assessed via self-report in the same instructional context, shared method variance and partial content overlap may have inflated associations. Accordingly, these results are best interpreted as evidence of strong covariation rather than definitive discriminant validity; future studies should confirm discriminant validity with multi-method designs and explicit measurement modeling.

### 4.6 Future research directions

Future work should prioritize stronger causal leverage. Cluster-randomized designs with more classes, active control conditions (e.g., non-aesthetic music activities or other arts-based courses), and longer follow-up windows would allow more confident conclusions about durability and specificity of effects.

To deepen mechanism testing, studies could combine self-report with behavioral tasks and physiological indicators (e.g., prosocial decision tasks, heart-rate variability, skin conductance) and examine whether changes in attention to expressive cues or emotion regulation mediate empathy gains. Neurocognitive methods (EEG/fMRI) may be useful for testing whether aesthetic judgment and empathic responding recruit overlapping processes under intervention conditions.

Finally, generalizability should be examined across different music genres, pedagogical models (listening-, creating-, or performing-focused curricula), age groups, and cultural contexts. Mixed-methods work (e.g., classroom observations and interviews) would help clarify how students experience aesthetic learning and how such experiences translate into empathic dispositions in everyday social settings.

## 5 Conclusion

This study examined whether an 8-week music aesthetic education course can promote empathy among Chinese undergraduate students and whether changes in aesthetic sensitivity (AS) help explain this association. Empathy increased significantly within the intervention class, whereas between-class post-test differences were positive but not uniformly statistically significant. AS showed clear pre–post gains in the intervention class and was positively associated with empathy in multilevel models that accounted for class nesting.

Mediation analyses were consistent with an indirect pathway through AS within the experimental class, supporting the interpretation that cultivating sensitivity to musical form, meaning, and emotional expression may be one pathway through which empathy gains emerge. At the same time, because the design relied on intact classes, the findings should be interpreted as supportive evidence rather than definitive proof of causality.

Overall, the results suggest that structured guided listening, reflection, and collaborative discussion can be a feasible approach for integrating aesthetic aims with empathy-related learning in higher education. Future replications with more classes, additional outcome measures, and longer follow-up are needed to confirm robustness and clarify boundary conditions.

## Data Availability

The original contributions presented in the study are included in the article/Supplementary material, further inquiries can be directed to the corresponding author.
