# Supplementary material for: Can heightened aesthetic sensitivity in music promote empathy?-A pre- and post-test multilevel linear model of an 8-week music aesthetic education course intervention
Source: Front Psychol. 2026 Apr 8;17:1744584. doi: 10.3389/fpsyg.2026.1744584 (PMC13099863; doi:10.3389/fpsyg.2026.1744584)
Supplement: Supplementary file 1 [file Supplementary_file_1.pdf]

Supplementary Appendix 1. 8-week course schedule

Weekly schedule of the music aesthetic education course, including course topics, learning objectives, main activities, and expected outcomes.

Week	Course Topic	Learning Objectives	Main Activities (Including Specific Work Examples)	Expected Outcomes
**1**	Introduction to Basic Musical Elements and Perception	Cultivate fine discrimination of musical microstructures (such as pitch, rhythm, dynamics), enhance the perceptual level of aesthetic sensitivity; initially understand music as a carrier of emotions and promote emotional contagion.	1. Introduce musical elements (pitch, rhythm, harmony, texture).<br>2. Listen to simple melody segments, such as the first movement of Beethoven's 'Moonlight Sonata' (perceive changes in dynamics and rhythm), and discuss the perceived changes in groups.<br>3. Personal reflection: record immediate emotional reactions triggered by music.<br>4. Experiential activity: simple rhythm tapping exercise to feel synchronization, using segments from the Chinese folk music 'Spring River Flower Moon Night'.	Students can identify basic musical elements and initially describe their impact on emotions; enhance sensitivity to emotional cues and form initial emotional resonance.
**2**	Musical Emotional Cues and Emotional Resonance	Deepen resonance with and regulation of musical emotional cues, enhance the ES dimension; through emotion-recognition exercises, promote primitive forms of emotional empathy (emotional contagion).	1. Appreciate music expressing different emotions, such as Mozart's 'Twinkle Twinkle Little Star Variations' (joyful) and Chopin's 'Nocturne in E-flat Major' (sad).<br>2. Analyze emotional arcs: group drawing to represent changes in musical emotions.<br>3. Discussion and sharing: how to 'receive' others' emotions in music (such as the composer's intent).<br>4. Cooperative activity: group imitation of emotional expression in simple chorus, using the Chinese traditional song 'Jasmine Flower'.	Students can identify and regulate emotional cues in music; initially experience emotional contagion, enhance ES, and connect it to empathy in interpersonal situations.
**3**	Musical Styles and Cultural Contexts	Train students to complete meaning construction within stylistic grammar and cultural contexts, enhance the evaluative dimension of aesthetic sensitivity; promote cognitive empathy through PT to understand multicultural emotions.	1. Introduce different cultural musical styles (such as Western classical, Chinese folk music, and African rhythms).<br>2. Comparative listening: analyze the influence of cultural backgrounds on musical expression, such as Tchaikovsky's 'Swan Lake' excerpt (Western ballet) and African drum music 'Djembe Rhythm'.<br>3. Group discussion: interpret work meanings from composer and cultural perspectives.<br>4. Experiential activity: appreciate cross-cultural music using digital performance tools, such as Indian raga music 'Raga Yaman', and record personal insights on cultural migration.	Students can construct musical meanings and adopt others' perspectives, enhancing cognitive empathy; understand cultural diversity and promote warm concern for others' emotions.
**4**	Aesthetic Experience and Emotional Regulation	Strengthen emotional mechanisms of aesthetic sensitivity (such as expectation violation and emotional contagion); through music-regulation exercises, enhance the empathetic-care level of emotional empathy.	1. Discuss aesthetic emotions (such as the sublime and compassion).<br>2. Listen to complex emotional music, such as the first movement of Beethoven's 'Symphony No. 5' (fate theme, expectation violation), and practice emotional regulation.<br>3. Role-playing: simulate the composer's emotions and share caring intentions.<br>4. Cooperative practice: group ensemble using Debussy's 'Clair de Lune' (Impressionist complex emotions).	Students can experience and regulate complex aesthetic emotions; enhance empathetic care, form action-oriented support for others, and improve overall emotional empathy.
**5**	Collaborative Musical Practice and Social Connections	Strengthen emotional resonance and PT through chorus and ensemble, enhance the social dimension of aesthetic sensitivity; promote interaction between cognitive and emotional empathy.	1. Group chorus training: focus on listening and responding to others, using Vivaldi's 'The Four Seasons: Spring' excerpt.<br>2. Ensemble activity: group performance of simple pieces, such as Bach's 'Minuet', and discussion of emotional synchronization in collaboration.<br>3. Reflection discussion: how to understand partners' intentions in music.<br>4. Performance preparation: integrate elements from previous weeks and simulate real performance scenarios, using the Chinese folk music 'High Mountains and Flowing Water'.	Students practice PT and emotional sharing in collaboration; enhance dynamic interaction of empathy and strengthen social-emotional abilities.
**6**	Musical Narrative and Meaning Interpretation	Deepen understanding of musical narratives, enhance the interpretive ability of aesthetic sensitivity; through narrative migration, promote viewpoint adoption in cognitive empathy.	1. Appreciate narrative music, such as Mussorgsky's 'Pictures at an Exhibition' (depicting exhibition scenes).<br>2. Analyze narrative emotional arcs and character emotions.<br>3. Group story creation: arrange narratives based on music and share others' perspectives, using Tchaikovsky's 'Sleeping Beauty' ballet excerpt.<br>4. Digital resource integration: use online performances to analyze cultural narratives, such as Wagner's opera 'The Ring of the Nibelung' excerpt.	Students can interpret musical meanings and transfer them to interpersonal narratives; enhance cognitive empathy and promote understanding and concern for others' experiences.

Week	Course Topic	Learning Objectives	Main Activities (Including Specific Work Examples)	Expected Outcomes
**7**	Comprehensive Aesthetic Reflection and Migration	Integrate various dimensions of aesthetic sensitivity, enhance evaluation and critical abilities; explore migration mechanisms from aesthetic experiences to empathy and promote action-oriented empathetic care.	1. Review music from previous weeks and comprehensively evaluate work values, such as comparing Beethoven's and Chopin's emotional expressions.<br>2. Reflection discussion: how aesthetic sensitivity affects daily empathy.<br>3. Case analysis: connect musical emotions with social situations (such as sad music 'Adagio for Strings' and others' anxiety).<br>4. Community collaboration: invite art clubs to share and expand practice boundaries, using contemporary music such as Joe Hisaishi's 'Spirited Away' soundtrack.	Students can critically reflect on aesthetic experiences; clarify the mediating role of aesthetic sensitivity in empathy and form intentions to transfer this concern to interpersonal contexts.
**8**	Performance Summary and Educational Extension	Consolidate the overall enhancement of aesthetic sensitivity and empathy; through performances, strengthen transfer and promote lasting emotional orientation and action guidance.	1. Student performance: group performance of self-created or adapted music incorporating emotional expression, such as self-created variations based on Mozart's variations.<br>2. Feedback discussion: adopt audience perspectives and share empathy experiences.<br>3. Summary reflection: record changes before and after the intervention and discuss the role of aesthetic education in personality shaping.<br>4. Outlook: suggest daily music practices to promote continuous development, using cross-cultural fusion works such as 'Silk Road Music'.	Students demonstrate comprehensive abilities, enhance confidence and empathy; deepen understanding of the educational function of music aesthetic education and form long-term aesthetic and empathy habits.
